# A Simple and Efficient Method of Removing Tonsils

**Published:** 1910-12-03

**Authors:** Douglas J. Guthrie


### A SIMPLE AND EFFICIENT METHOD OF REMOVING TONSILS.

By DOUGLAS J. GUTHRIE, M.D. Edin.

There is probably no operation more frequently undertaken by the general practitioner than the removal of enlarged tonsils, and none when properly performed gives such excellent results. But it is not by any means an uncommon experience to meet with cases in which the manipulations have been imperfectly carried out, so that the patient is not benefited. Doubtless it is partly on this account that within recent times enucleation, that is to say removal of the entire tonsil in its surrounding capsule, has been strongly advocated by some as the operation of choice. Like all new forms of treatment it may run the risk of being employed to the exclusion of older methods, unless we bear in mind that it has its limitations as well as its uses.

Whilst it is unlikely that enucleation, in preference to the ordinary tonsillotomy, will ever become the routine method of dealing with enlarged tonsils, it is undoubtedly the best treatment for certain selected cases. In adults, coming under our notice as they so often do on account of constantly recurring quinsy, anything short of total removal of the area of infection must be regarded as unsound in principle, and this is borne out by the fact that we not infrequently find quinsies in persons whose tonsils have already been partly removed in the usual way. Hence it might almost be laid down as a general working rule that if the patient be an adult enucleation is to be preferred, the tonsil being so often the seat of disease, rather than mere hypertrophy, in such cases. Enucleation is superior to the slower and more laborious mode of reducing the tissue by means of punch forceps and cautery, although this treatment may suggest itself as an alternative, should time be no object to the patient.

With children the case is different; one is often called upon to remove tonsils with or without coexistent adenoids, merely on account of the obstruction to breathing and alteration of the voice caused thereby, and complete removal is consequently not essential. But when it is certain that the tonsil is diseased, as when it constitutes a source of lymphatic gland infection, or gives rise to septic sore-throat at frequent intervals, enucleation is the correct treatment. Further, enucleation is the only method for those cases in which the affected tonsil projects little, if at all, beyond the pillars of the fauces, the so-called flat or sessile tonsil.

Passing to the technique of the operation, there are several facts which must ever be borne in mind if success is to be attained. And first of all it may be said that there is no tonsil which the guillotine, properly employed, will fail to remove. Various ways of enucleating have been recommended, and special instruments constructed for dissecting the tonsil out of its bed, but the guillotine method is simplicity itself, and involves no elaborate dissection. A Mackenzie's or a Heath's instrument will be found most useful, the two essentials being that it need possess no fork and, what is of much greater importance, the size of the ring should be slighly smaller than the tonsil we are about to remove. Inattention to this point is the chief source of failure to effect a complete excision of the tonsil. I have found guillotine No. 1 or No. 2 most serviceable, No. 3 is occassionally required, No. 4 seldom or never.

The operation is performed under local or general anæsthesia. It is much easier of execution when a local anæsthetic is used, but if, as in case of children, unconsciousness is essential, chloroform or A.C.E. should be employed. Ethyl chloride does not allow sufficient time for a satisfactory enucleation. As a preliminary step the tonsil should, if necessary, be freed from the anterior pillar by a few touches of the scalpel. It is then firmly grasped by vulsellum forceps, which have been introduced through the ring of the guillotine. The ring is next passed over the tonsil and pressed firmly outwards, the vulsellum in the operator's left hand pulling the tonsil forcibly inwards, whilst the blade of the guillotine is driven home. As division is effected, both the vulsellum and the shaft of the guillotine should be directed towards the tonsil from the opposite angle of the mouth. Particular care should be taken to remove the lower pole of the tonsil, a part hidden from view and hence liable to be left behind. In many cases it will be found easier to grasp the tonsil after engaging it in the ring of the guillotine, whilst for this purpose one or other of the specially devised tonsil forceps may be used. If the manipulations have been carefully carried out, always under the direct guidance of the eye, it will be found that the tonsil has been removed in its capsule, leaving behind a smooth-walled cavity, from which hæmorrhage rarely takes place, and whose raw surface granulates and heals very rapidly. Hæmorrhage of any severity is much less frequent than in tonsillotomy. Confinement to bed is unnecessary, and the patient may in many instances return to work on the following day.

